# Fungal metabolic profile dataset was not influenced by long-term *in vitro* preservation of strains

**DOI:** 10.1016/j.dib.2019.104568

**Published:** 2019-09-26

**Authors:** Tereza Veselská, Miroslav Kolařík

**Affiliations:** a Laboratory of Fungal Genetics and Metabolism, Institute of Microbiology, Czech Academy of Sciences (CAS), Vídeňská 1083, CZ-14220 Prague, Czech Republic; b Department of Botany, Faculty of Science, Charles University, Benátská 2, CZ-12801 Prague, Czech Republic

**Keywords:** Fungi, Metabolic profile, Biolog microarray, Fungal physiology, *In vitro* preservation, Comparative ecophysiology

## Abstract

Comparative ecophysiology is highly valuable approach to reveal adaptive traits linked with specific ecological niches. Although long-term *in vitro* preserved fungal isolates are often used for analyses, only sparse data is available about the effect of such handling on fungal physiology. The purpose of our data is to show the effect of long-term *in vitro* preservation of fungal strains on their metabolic profiles. This data is related to research paper “Adaptive traits of bark and ambrosia beetle-associated fungi” (Veselská et al., 2019). Biolog MicroPlates™ for Filamentous fungi were used to compare metabolic profiles between freshly isolated and long-term *in vitro* preserved strains of two *Geosmithia* species. Additionally, carbon utilization profiles of 35 *Geosmithia* species were assessed, including plant pathogen *G. morbida* and three ambrosia species. Data also shows differences in carbon utilization profiles among diverse ecology types presented in the genus *Geosmithia*.

**Table:** Specifications Table

Subject area	*Microbiology*
More specific subject area	*Fungal physiology*
Type of data	*Table, graph*
How data was acquired	*Biolog MicroPlate*^*TM*^*for Filamentous fungi, plate reader INFINITE M2*00 TECAN *(Tecan Instrument, Austria) with MAGELLAN software, PAST program*
Data format	*Analyzed data, Raw data in supplementary material*
Experimental factors	*Species ecology and time of preservation, i.e. short vs. long-term.*
Experimental features	*Fungal conidia were inoculated into Biolog MicroPlates*^*TM*^*for Filamentous fungi and the absorbance at* 750 nm *was recorded to assess fungal growth. Comparative ecophysiology and comparison of freshly isolated and long-term in vitro preserved fungal strains were assessed using statistical program PAST.*
Data source location	*Collection location, plant and beetle hosts are in Table 1*
Data accessibility	*Data is with this article.*
Related research article	*Veselská, T., Skelton, J., Kostovčík, M., Hulcr, J., Baldrian, P., Chudíčková, M., Cajthaml, T., Vojtová, T., Garcia-Fraile, P. and Kolařík, M., 2019. Adaptive traits of bark and ambrosia beetle-associated fungi. Fungal Ecology. 41, 165–176.*https://doi.org/10.1016/j.funeco.2019.06.005.

**Table:** 

**Value of the Data** • Comparative ecophysiology is valuable tool for tracing of species adaptive traits and identification of potential virulence factors in plant, animal and human pathogenic fungi. Usually, long-term *in vitro* preserved isolates are used for physiological analysis, but little is known about the effect of such handling on fungal physiology. Presented data investigate the reliability of using the long-term preserved fungal cultures for physiological analysis. • Data disproves negative effect of long-term preservation on fungal metabolic profile, which enables researchers to use such strains for physiological studies. • Data shows metabolic profiles of carbon utilization for most of *Geosmithia* species which includes also ambrosia fungi and severe phytopathogen *G. morbida.* • Raw data provides growth values on each carbon source. This is helpful for further identification of adaptive traits of these important species.

## 1 Data

**Fig. 1:**
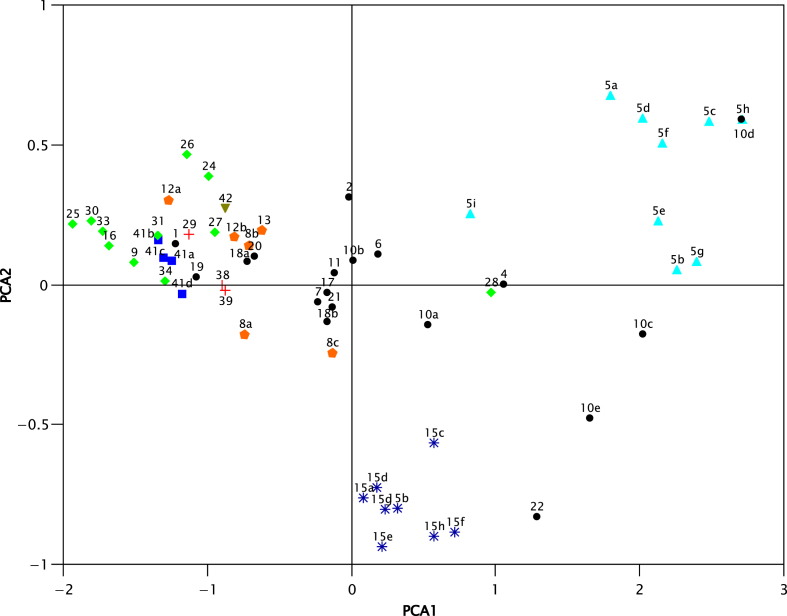
Principal component analysis (PCA) plot of the metabolic profiles of 60 *Geosmithia* strains and comparison of “new” and “old” strains of *G.* sp. 5 and *G. langdonii*. Different ecology types as follow: diamond – long-term co-evolved specialists, dot, triangle, star – facultative symbionts, cross – obligatory symbiont, inverted triangle – auxiliary ambrosial fungi, polygon, square – hardwood specialists, square – pathogen, triangle – new (5a-f) and old (5g-i) strains of *G.* sp. 5, star – new (15a-d) and old (15e-h) strains of *G. langdonii*. Based on one-way NPMANOVA, facultative generalists were significantly (p < 0.005) different from long-term co-evolved specialists and phytopathogen.

Biolog MicroPlate™ for Filamentous fungi was used to assess carbon sources utilization profiles of *Geosmithia* fungi living in symbiosis with bark beetles [1]. Their ecology spans from facultative to obligatory ambrosia symbiosis and from saprotrophic to pathogenic nourishment of severe phytopathogen *G. morbida* (Table 1). The aims were to test whether metabolic profiles of *Geosmithia* species are modified by their ecology and whether long-term preservation of strains has effect on their metabolic profiles. The distinct metabolic profiles belonging to particular ecology types are pictured in Fig. 1 and Table S1. The similarity in metabolic profiles of freshly isolated and long-term preserved strains of *Geosmithia* sp. 5 and *G. langdonii* is shown in Fig. 1 and Table S1. Raw data containing growth value of individual strains on each carbon source is presented in Table S1. Raw data is helpful for further identification of adaptive traits of important ambrosia and pathogenic species.

**Table 1:** List of *Geosmithia* species.

Species	Ecology type	Strain code	Culture collection	Strain code in Fig. 1	Substrate (mostly as insect vector/plant hosts)	Locality	Year of isolation	Reference
*G.* sp. 1	PF, G	1_1790	CCF4529	1	*Hypoborus ficus/Ficus carica*	Azerbaijan, Shaki Rayonu	2006	[6]
*G.* sp. 2	PF, G	2_1510	CCF4270	2	*Scolytus kirschii/Ulmus minor*	Italy, Termoli	2004	[6]
*G.* sp. 4	PF, G	4_1722	CCF4278	4	*Pteleobius vittatus F./Ulmus laevis*	Czech R., Břeclav	2004	[7]
*G. putterillii*	PF, G	6_103	CCF3342	6	*Scolytus rugulosus*/*Prunus* sp.	Czech R., Velemín	2000	[8]
*G. flava*	PF, G	7_264	CCF3354	7	*Hylesinus fraxini/Fraxinus excelsior*	Slovakia, Muráň castle	2002	[8]
*G.* sp. 8	PF, HWS	8_124	CCF3350	8a	*Scolytus intricatus/Quercus* sp.	Czech R., Prague	2001	[7]
		8_1712a	CCF4277	8b	*Scolytus intricatus/Quercus cerris*	Bulgaria, Kardzaly	2005	[7]
		37_1806	CCF4207	8c	*Scolytid beatle/Acacia smithii*	Australia, Eungella, Credition Hall	2006	[6]
*G.* sp. 11	PF, G	11_551	CCF3555	11	*Scolytus intricatus/Quercus pubescens*	Hungary, Vilányi hegy Mts.	2003	[7]
*G.* sp. 12	PF, HWS	12_284	CCF4300	12a	*Ernoporicus fagi/Fagus silvatica*	Slovakia, Pieniny National Park	2002	[7]
		12_1632	CCF4274	12b	*Hylesinus varius/Fraxinus excelsior*	Czech R., Pacov	2005	[7]
*G. ulmacea*	PF, HWS	13_924	CCF4601	13	*Scolytus multistriatus/Ulmus minor*	Czech R., Hodonín, Bulhary	2004	[7]
*G. obscura*	PF, G	17_391	CCF3424	17	*Taphrorychus bicolor/Fagus sylvatica*	Czech R., Louny, Hřivice	2003	[7]
*G. lavendula*	PF, G	18_1219	CCF4268	18a	*Hypoborus ficus/Ficus carica*	Croatia, Dalmatia, Sibenik	2005	[6]
		18_1781	CCF4285	18b	*Hypoborus ficus/Ficus carica*	Azerbaijan, Baki Sahari, Baku	2006	[6]
*G.* sp. 19	PF, G	19_1085a	CCF3658	19	*Hypoborus ficus/Ficus carica*	Italy, Molise, Termoli	2004	[6]
*G.* sp. 20	PF, G	20_764	CCF4527	20	*Phloetribus scarabeoides/Olea europea*	Syria, Krak des Chevaliers	2004	[6]
*G.* sp. 21	PF, G	21_1665	CCF4530	21	*Hypoborus ficus/Ficus carica*	Spain, Rosal de la Frontera	2005	[6]
*G.* sp. 22	PF, G	22_739	CCF3645	22	*Phloetribus scarabeoides/Olea europea*	Jordan, Wadi al Mujib	2004	[6]
*G. morbida*	HWS, P	41_1218	CCF3879 (CBS 124664)	41a	*Pityophthorus juglandis/J. nigra*	USA, Colorado, Boulder	2007	[9]
		41_U173	CCF4576	41b	*Pityophthorus juglandis/J. nigra*	USA, California, Rio Oso	2009	[9]
		41_U1259.55	–	41c	*Pityophthorus juglandis/Juglans* sp.	USA, Oregon	2008	[9]
		41_U1259.59	–	41d	*Pityophthorus juglandis/Juglans* sp.	USA, Oregon	2008	[9]
*G.* sp. 9	PF, SP	9_1210	CCF3703	9	*Cryphalus piceae/Abies alba*	Poland, Myślenice	2005	[10]
*G.* sp. 16	PF, SP	16_08 m	CCF4201	16	Pityophthorus pityographus/Picea abies	Poland, Czajowice	2007	[11]
*G.* sp. 24	PF, SP	24_RJ06ka	CCF4525	24	*Pityogenes bidentatus/Pinus sylvestris*	Poland, Zaborze	2007	[11]
*G.* sp. 26	PF, SP	26_1796	CCF4223	26	*Pityophthorus pityographus/Pinus silvestris*	Czech R., Seník	2006	[11]
*G.* sp. 27	PF, SP	27_0919	CCF4206	27	*Pityogenes bidentatus/Pinus silvestris*	Poland, Żurada	2006	[11]
*G.* sp. 28	PF, SP	28_279	CCF4210	28	*Polygraphus poligraphus/Picea abies*	Poland, Chyszówki	2007	[11]
*G.* sp. 30	PF, SP	30_09 m	CCF4209	30	*Pityophthorus pityographus/Picea abies*	Poland, Czajowice	2007	[11]
*G.* sp. 31	PF, SP	31_21k	CCF4526	31	*Pityophthorus pityographus/Pinus sylivestris*	Poland, Czajowice	2007	[11]
*G.* sp. 29	PF, SP	33_1827b	CCF4221	33	*Pityophthorus pityographus + Cryphalus piceae/Abies alba*	Czech R., Boubín hill	2008	[11]
*G.* sp. 30	PF, SP	34_1833	CCF4208	34	*Cryphalus abietis/Abies alba*	Czech R., Jílové u Prahy	2008	[11]
*G.* sp. 25	PF, SP	35_1835	CCF4205	25	*C. piceae + P. pityographus/Abies alba*	Czech R., Plešné jezero lake	2008	[11]
*G.* sp. 5	PF, G	5_U1.2c.25	CNR28	5a	*Scolytus multistriatus/Ulmus minor*	Czech R., Středokluky	2009	[2]
		5_U6.3e.35	CNR48	5b	*Scolytus multistriatus/Ulmus minor*	Czech R., Velký Osek	2009	[2]
		5_U7.8b	CNR30	5c	*Scolytus multistriatus/Ulmus laevis*	Czech R., Velký Osek	2009	[2]
		5_U8.1a	CNR49	5d	*Scolytus multistriatus/Ulmus minor*	Czech R., Maršovice	2009	[2]
		5_U8.1b	–	5e	*Scolytus multistriatus/Ulmus minor*	Czech R., Maršovice	2009	[2]
		5_U8.12b	–	5f	*Scolytus multistriatus/Ulmus minor*	Czech R., Maršovice	2009	[2]
		5_580	–	5g	*Hypoborus ficus/Ficus carica*	France, Biaritz, Ondres	2003	[6]
		5_1550	CCF4271	5h	*Scolytus intricatus/Quercus petraea*	Czech R., Mlynářův luh, 1997	1997	[7]
		5_137 m	CCF4215	5i	*Pityophthorus pityographus galleries/Picea abies*	Poland, Szydłowiec	2007	[11]
*G. omnicola*	PF, G	10_989	CCF3560	10a	*Scolytus pygmaeus/Ulmus minor*	Czech R., Břeclav	2004	[7]
		10_1788	CCF4286	10b	*Hypoborus ficus/Ficus carica*	Azerbaijan, Suvalan	2006	[6]
		10_U2.6a	CNR5	10c	*Scolytus multistriatus/Ulmus minor*	Czech R., Středokluky	2009	[2]
		10_U7.5a	CNR8	10d	*Scolytus multistriatus/Ulmus laevis*	Czech R., Velký Osek	2009	[2]
		10_942	–	10e	*Hypoborus ficus/Ficus carica*	Croatia, Brač Island	2004	[6]
*G. langdonii*	PF, G	15_U5.3a	CNR11	15a	*Scolytus multistriatus/Ulmus minor*	Czech R., Velký Osek	2009	[2]
		15_U7.9a	CNR6	15b	*Scolytus multistriatus/Ulmus laevis*	Czech R., Velký Osek	2009	[2]
		15_U8.6c	CNR117	15c	*Scolytus multistriatus/Ulmus minor*	Czech R., Maršovice	2009	[2]
		15_U8.12a	–	15d	*Scolytus multistriatus/Ulmus minor*	Czech R., Maršovice	2009	[2]
		15_1645	–	15e	*Scolytus multistriatus/Ulmus laevis*	Czech R., Neratovice	2005	[12]
		15_1683	CCF4276	15f	*Ernoporus tiliae/Tilia* sp.	Czech R., Nové Hrady	2005	[7]
		15_1603c	CCF3562	15g	*Phloeosinus thujae/Thuja occidentalis*	Czech R., Poříčí nad Sázavou	2005	[7]
		15_1619	CCF4272	15h	*bostrichid beetle/Pistacia lentiscus*	Portugal, Sesimbra	2005	[6]
*G. cnesini*	AF	29_1820	CCF4292	29	*Cnesinus lecontei/Croton draco*	Costa Rica, Heredia	2007	[13]
*G. microcorthyli*	AF	38_A2	CCF3861	38	*Microcorthylus* sp.*/Cassia grandis*	Costa Rica, Heredia	2006	[14]
*G. eupagioceri*	AF	39_A1	CCF3754	39	*Eupagiocerus dentipes/Paullinia renesii*	Costa Rica, Heredia	2006	[14]
*G. rufescencs*	AAF	42_1821	CCF4524	42	*Cnesinus lecontei/Croton draco*	Costa Rica, Heredia	2007	[14]

Ecology: PF – association with phloem feeding beetles, G – generalist, SF – specialists to *Fagus*, SP – specialist to Pinaceae, HWS – hardwood specialists, P – pathogen, AF –ambrosia fungi, AAF – auxiliary ambrosia fungi.

## 2 Experimental design, materials and methods

### 2.1 Fungal strains

The metabolic profiles of 60 strains belonging to 35 *Geosmithia* species (Table 1) were analyzed. These strains are deposited in the Culture Collection of Fungi (CCF) or at Institute of Microbiology of the Czech Academy of Sciences for several years. Then, two species, *G.* sp. 5 and *G. langdonii,* were chosen and the effect of long-term *in vitro* preservation (0–10 years) on fungal carbon assimilation profiles was observed. Fresh strains of these species were isolated from active beetle galleries in 2009 and identified as it is described in Pepori et al. [2]. These strains were analyzed within a 2 months on Biolog MicroPlates™ for Filamentous fungi. Altogether, three “old” and six “new” strains of *G.* sp. 5 and four “old” and four “new” strains of *G. langdonii* were compared. The species classification follows Kolařík et al. [3].

### 2.2 Biolog MicroPlate™ for Filamentous fungi

Biolog MicroPlate™ for Filamentous fungi contains 95 different dried carbon sources and one negative control. Fungal conidia from grown cultures were transferred into the inoculating fluid (0.25% Phytagel, 0.03% Tween 40) by rolling a swab across sporulating areas to get the final transmittance of 75 ± 2%. The inoculated plates (200 μl per well) were then incubated in the dark at 25 °C and absorbance at 750 nm was used to measure mycelial growth at 24, 48, 72, 96 and 168 h. An absorbance reading taken 96 h after the inoculation was included in the analysis, because sporulation occurred in some strains after that time. Two technical replicates per strain were prepared.

### 2.3 Statistical analysis

The absorbance of the negative control was subtracted from all substrates within one plate and negative values were assigned a value of zero [4]. Biolog™ data were visualized on PCA (Principal Component Analysis) in PAST program [5]. The statistical significance of the type of ecology was evaluated by one-way NPMANOVA with Bonferroni-corrected *p* values using Bray-Curtis distance and 9999 permutations.
